# Successful Cardiac, Lung, and Kidney Transplantation from a Methanol-poisoned Donor

**DOI:** 10.31662/jmaj.2023-0081

**Published:** 2023-12-11

**Authors:** Takashi Hongo, Tetsuya Yumoto, Yoshinori Kosaki, Tomohiro Hiraoka, Kohei Tsukahara, Tsuyoshi Nojima, Takafumi Obara, Kohei Ageta, Yukie Yamasaki, Kaori Taniguchi, Masanobu Miura, Satoru Miyaishi, Hiromichi Naito, Atsunori Nakao

**Affiliations:** 1 Department of Emergency, Critical Care, and Disaster Medicine, Okayama University Graduate School of Medicine, Dentistry, and Pharmaceutical Sciences, Okayama, Japan; 2 Department of Legal Medicine, Okayama University Graduate School of Medicine, Dentistry, and Pharmaceutical Sciences, Okayama, Japan; 3 Department of Legal Medicine, Kawasaki Medical School, Kurashiki, Japan

**Keywords:** brain death, methanol, transplantation, donor

## Abstract

Massive methanol exposure can lead to severe and detrimental effects that can result in death or brain death. As organs from patients with brain death after methanol ingestion are less likely to be recovered, these patients have been considered marginal donors. We present a case of successful multiple organ transplantation (heart, lungs, and kidneys) from a methanol-poisoned patient. Our experience illustrates that donor death from methanol intoxication does not preclude organ transplantation.

## Introduction

Methanol poisoning, while uncommon, can cause brain death and irreversible loss of neurological function ^(1)^. Given the global shortage of organ donors and the growing waiting lists for transplantation, investigations are required to increase available donor pools ^(2)^. Historically, patients succumbing to various types of poisoning were not considered acceptable for organ donation ^(3)^. However, accumulating evidence is surfacing in the literature regarding the use of organs from patients dying from intoxication by certain medicines, drugs, and industrial and domestic products as donors ^(4)^. They may represent 1% of all organ donors and comprise 1.1% of all organs transplanted ^(3), (5)^.

Herein, we report the successful outcome involving heart, lung, and kidney transplantation from a methanol-poisoned donor. Our case, as along with previous reports, indicated that methanol exposure is not an absolute contraindication for organ transplantation ^(5)^. This is the first case report of organ donation from a methanol-poisoned cadaveric donor in Japan.

## Case Report

**Figure 1.**
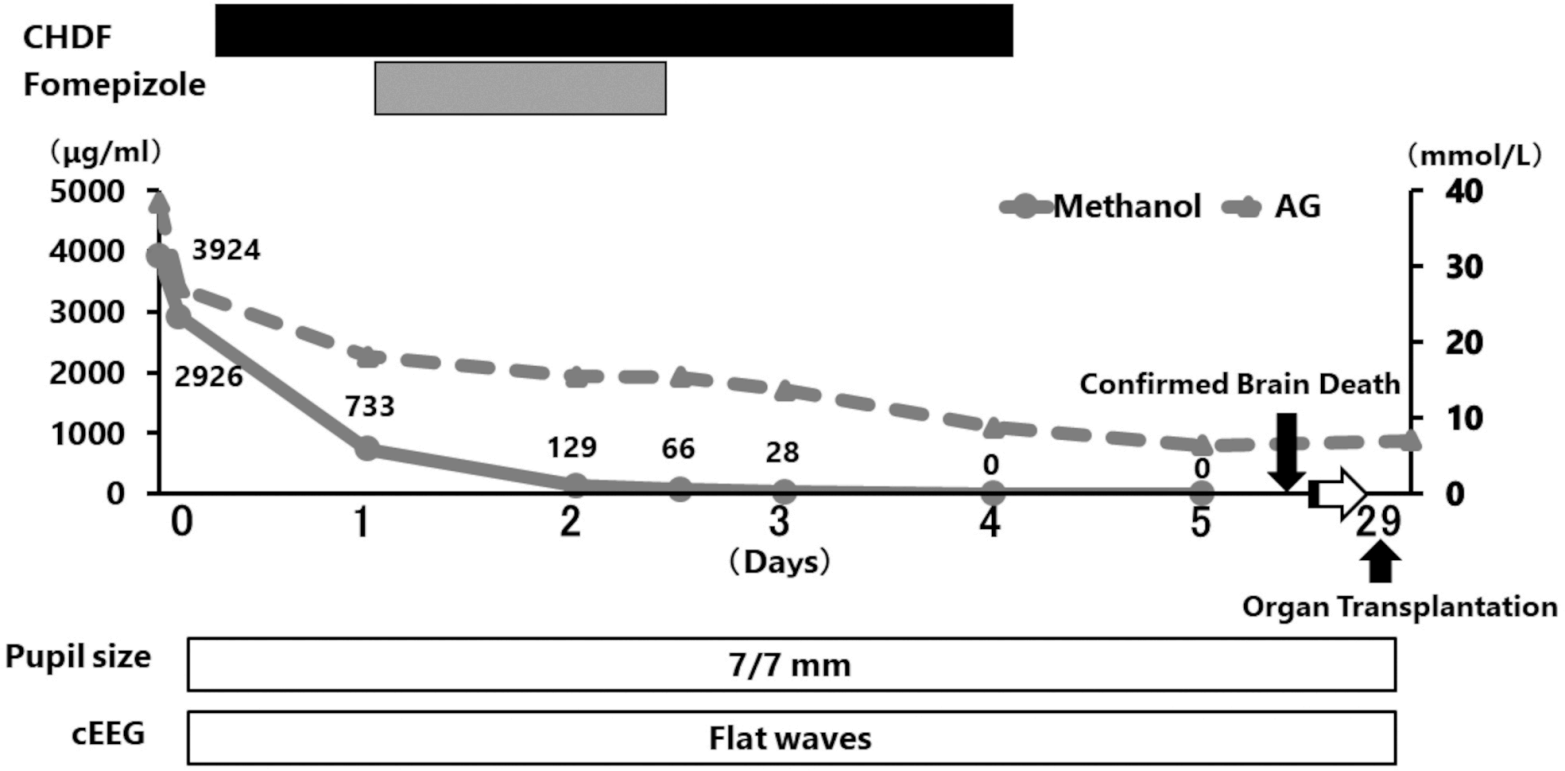
Patient’s clinical course. AG, Anion Gap; CHDF, continuous hemodiafiltration; cEEG, continuous electroencephalogram.

A 35-year-old man was taken to our emergency department due to impaired consciousness. Upon arrival, his Glasgow Coma Scale score was 3 points (E1V1M1) with bilateral pupils dilated. His vital signs were as follows: blood pressure of 140/115 mmHg, heart rate of 136/min, respiratory rate of 26/min, and body temperature of 36.5°C. Blood gas analysis revealed severe metabolic acidosis with a pH of 6.821, lactate level of 11.2 mmol/L, and anion gap of 38.5 mmol/L. His measured and calculated serum osmolality was 389 and 313 mOsmol/kg, respectively. Computed tomography demonstrated pancreatolithiasis and chronic alcoholic changes without ascites. His family and colleagues testified that the patient drank hidden alcohol-based hand sanitizers containing 58% methanol. Moreover, the family stated that the patient had a chronic habit of consuming alcohol and sometimes exhibited alcohol-seeking behaviors. At the time of admission, the blood concentration of methanol was 3,942 μg/ml, which was considered lethal (Figure 1). Based on the examination results and the episode of methanol ingestion, a clinical diagnosis of metabolic acidosis due to methanol intoxication was made. Hemodialysis for methanol/formic acid elimination and oral fomepizole administration were initiated in addition to supportive therapy, including the infusion of sodium bicarbonate solution. Sequential measurement of blood methanol showed a gradual decrease in methanol at 24 h and achieved a plasma methanol concentration of zero at 96 h.

Despite aggressive intervention, neurological examination revealed a flat electroencephalogram and an absent auditory brainstem response at 177 h after methanol ingestion. Based on the evidence of brain death, the possibility of organ donation was discussed, as metabolic disorders were corrected and methanol had disappeared from the blood (Figure 1). After obtaining family consent for organ donation, the heart, kidneys, and lungs were retrieved. Procurement of the liver and pancreas was refused by the patient’s family. During the procurement procedure, all organs were certified to have a normal appearance. All recipients uneventfully recovered during the 3-month follow-up period.

## Discussion

The primary concern lies in insufficient knowledge regarding the organ-specific toxicity of methanol, resulting in premature exclusion of organs for procurement. Concerns may exist regarding whether the organs of the donor subjected to extraction for transplantation contain the toxic byproducts of methanol, causing intoxication in the recipients of these grafts. In autopsy cases of methanol poisoning, high concentrations of methanol and its toxic metabolites have been found in the kidney, liver, and gastrointestinal tract ^(6)^. However, methanol is not listed as a drug with significant organ concentrations on which the reservoir effect may occur after implantation into the recipients ^(4)^. Although this carryover could be particularly important with liposoluble drugs and toxins that accumulate preferably in the organs subject to transplantation, previous literature has demonstrated that methanol is not transferred from the donor to the recipient ^(3)^.

In our review of the literature from the past 20 years, we found that 49 kidneys, 1 heart, 1 liver, 1 lung, and 2 corneal transplants were performed. Most of them indicated favorable long-term graft function and recipient survival (Table 1) ^(7), (8), (9), (10), (11), (12), (13), (14), (15)^. Although available data including the follow-up period are limited, it is most likely that the survival of organ grafts procured from donors who died due to methanol does not differ in the short- and long-term from transplants performed with organs from donors who died from other causes ^(16)^.

**Table 1.** Organ Transplantation from Methanol-Poisoned Donors in the Medical Literature.

Author	Year	Number of donors	transplanted organs	Follow-up
Stoiber L, et al ^(7)^	2021	1	1 heart	Alive at 32 months after transplantation with normal cardiac function.
Zomorrodi A, et al ^(8)^	2020	1	2 kidneys	Alive at 3 weeks after transplantation with normal renal function.
Sklienka P, et al ^(9)^	2014	3	6 kidneys	Of 3 patients, 2 were alive at 3 months posttransplantation with normal renal function, and 1 was alive at 4 months with graft dysfunction.
Gunka I, et al ^(10)^	2013	6	12 kidneys	Of 6 patients, 1 died and 1 experienced graft dysfunction.
Klimaszyk D, et al ^(11)^	2013	1	2 corneal	Not available
Kolaciński Z, et al ^(12)^	2013	13	24 kidneys	All patients were alive at 18 months after transplantation with normal renal function.
Silva JA et al ^(13)^	2004	1	1 lung and 1 kidney	Not available
Duque E, et al ^(14)^	2004	1	2 kidneys	Not available
Zota V, et al ^(15)^	2003	1	1liver and 2 kidneys	Alive at 5 months after transplantation with normal renal and hepatic function.

Medical professionals belonging to transplantation centers should discuss this in collaboration with clinical toxicologists and collect more information on the referred poisoned patients who are not accepted as suitable donor candidates.

## Conclusion

Our experience, along with previous reports, demonstrated that a methanol-poisoned donor is not a contraindication for organ transplantation.

## Article Information

### Conflicts of Interest

None

### Acknowledgement

We thank Christine Burr for editing the manuscript.

### Author Contributions

All authors meet the ICMJE authorship criteria.

YY, KT, MM, and SM: acquisition, analysis, and interpretation of data; YK, TH, KT, TN, TO, and KA: in charge of the patient in our hospital; TH, TY, HN, and AN: conception and design of the study, analysis, and interpretation of data, and drafting or revision of the manuscript. The other authors have made substantial revisions and edits. All authors have read and approved the final manuscript.

### Approval by Institutional Review Board (IRB)

Not applicable.

### Informed Consent

Written informed consent was obtained from the patient’s family.

### Registry and Registration no. of the Study/Trial

Not applicable.

## References

[1] Fraser AD. Methanol poisoning. CMAJ. 1993;149(2):134-6. PMC1485413 8324702

[2] Bentley MJ, Mullen JC, Lopushinsky SR, et al. Successful cardiac transplantation with methanol or carbon monoxide-poisoned donors. Ann Thorac Surg. 2001;71(4):1194-7. 11308158 10.1016/s0003-4975(01)02402-x

[3] López-Navidad A, Caballero F, González-Segura C, et al. Short- and long-term success of organs transplanted from acute methanol poisoned donors. Clin Transplant. 2002;16(3):151-62. 12010136 10.1034/j.1399-0012.2002.01109.x

[4] Leikin JB, Heyn-Lamb R, Aks S, et al. The toxic patient as a potential organ donor. Am J Emerg Med. 1994;12(2):151-4. 8161385 10.1016/0735-6757(94)90235-6

[5] Hantson P, Mahieu P. Organ donation after fatal poisoning. QJM. 1999;92(7):415-8. 10627892 10.1093/qjmed/92.7.415

[6] Mittal BV, Desai AP, Khade KR. Methyl alcohol poisoning: an autopsy study of 28 cases. J Postgrad Med. 1991;37(1):9-13. 1941700

[7] Stoiber L, Schoenrath F, Knosalla C, et al. Case report: Early transplant rejection of a methanol-intoxicated donor heart in a young female patient. A diagnostic approach with CMR, cardiac biopsy, and genetic risk assessment. Front Immunol. 2021;11:575635. 33692775 10.3389/fimmu.2020.575635 PMC7938323

[8] Zomorrodi A, Kakaei F. Successful kidney transplant from a brain stem-dead donor due to lethal methanol poisoning. Exp Clin Transplant. 2020;18(7):832-3. 31615377 10.6002/ect.2019.0238

[9] Sklienka P, Neiser J, Sevcík P, et al. Successful kidney transplant from methanol-intoxicated donors. Prog Transplant. 2014;24(2):199-205. 24919738 10.7182/pit2014111

[10] Gunka I, Samlík J, Mazur M, et al. Kidney transplantation from donors dying of methanol intoxication. Rozhl Chir. 2013;92(4):201-4. 23965006

[11] Klimaszyk D, Paciorek P, Wieczorek J. Corneal donation from a victim of methanol poisoning--Case report. Przegl Lek. 2013;70(8):679-80. 24466719

[12] Kolaciński Z, Skrzypek-Mikulska A, Pitrus E, et al. Kidney transplants from donors burdened metabolic acidosis in the course of poisoning with methanol and carbon monoxide. Przegl Lek. 2013;70(8):514-9. 24466684

[13] Silva JA, Chamorro C, Varela A, et al. Successful bilateral lung transplantation from a methanol-poisoned donor. Transplant Proc. 2004;36(9):2806-7. 15621154 10.1016/j.transproceed.2004.09.054

[14] Duque E, Duque J, Henao J, et al. Organs transplanted from intoxicated donors. Transplant Proc. 2004;36(6):1632-3. 15350437 10.1016/j.transproceed.2004.06.017

[15] Zota V, Popescu I, Ciurea S, et al. Successful use of the liver of a methanol-poisoned, brain-dead organ donor. Transpl Int. 2003;16(6):444-6. 12819879 10.1007/s00147-003-0559-5

[16] Wood DM, Dargan PI, Jones AL. Poisoned patients as potential organ donors: postal survey of transplant centres and intensive care units. Crit Care. 2003;7(2):147-54. 12720561 10.1186/cc1880 PMC270623

